# Supplementary material for: Complexome profiling of the Chlamydomonas psb28 mutant reveals TEF5 as an early PSII assembly factor
Source: Plant Cell. 2025 Mar 17;37(6):koaf055. doi: 10.1093/plcell/koaf055 (PMC12164590; doi:10.1093/plcell/koaf055)
Supplement: koaf055_Supplementary_Data [file koaf055_supplementary_data.zip › psb28_interactive.zip!/plots/intensity/qualityPlots/peptides/MAW6_Cre07.g321400.t1.1.html]

### Cre07.g321400.t1.1

Trivial name: MAW6  
SeqCoverage [median]: 10.00  
Mass: 199.24 kDa  
  
 

| Sequence | MissedCleavage | Mass [Da] | wtIntensity | muIntensity | Score |
| --- | --- | --- | --- | --- | --- |
| ADIINYVNK | 0 | 1048.56 | 829.11 | 860.71 | 88.94 |
| AFYGQAYR | 0 | 974.46 | 5774.82 | 3710.87 | 103.29 |
| DAGSSVNSNTLR | 0 | 1219.58 | 9165.13 | 10350.52 | 176.95 |
| DTGATYR | 0 | 782.36 | 2862.16 | 1374.65 | 116.30 |
| GGSMVVLTQAGFGK | 0 | 1350.70 | 4981.52 | 6333.20 | 155.00 |
| GVLALQTIAVTNQDTSGNTVTVGK | 0 | 2386.27 | 691.45 | 3907.10 | 61.96 |
| LESDPTVTYK | 0 | 1151.57 | 3259.85 | 3364.45 | 115.71 |
| MLYIPSNDLNTPGGISSTLNK | 0 | 2234.13 | 1624.57 | 1373.66 | 93.68 |
| NSIEGWVASANYPVSK | 0 | 1720.84 | 0.00 | 873.52 | 90.44 |
| SDNVALTGYYVTIPFK | 0 | 1786.91 | 878.59 | 0.00 | 155.66 |
| SGSAPVAGVSTY | 0 | 1094.52 | 1669.77 | 1482.35 | 168.27 |
| SPVTMVGR | 0 | 845.44 | 10282.33 | 10162.74 | 146.54 |
| STNIITIK | 0 | 888.53 | 3348.61 | 5918.49 | 99.44 |
| STNIITIKDTGATYR | 1 | 1652.87 | 562.19 | 1154.31 | 56.48 |
| VLATQAR | 0 | 757.44 | 19581.74 | 16513.07 | 136.89 |
| VSINEVSK | 0 | 874.48 | 10871.94 | 9705.35 | 128.12 |
| VSTTPTVSTK | 0 | 1019.55 | 13985.11 | 11925.52 | 99.80 |
|  |  |  | 90368.9 | 89010.5 |  |
